# Prevalence and pollution characteristics of antibiotic resistant genes in one high anthropogenically-impacted river

**DOI:** 10.1371/journal.pone.0231128

**Published:** 2020-04-09

**Authors:** Qingzhao Li, Qiuling Zhang

**Affiliations:** 1 Research Center of Environment Pollution Control and Restoration, Zhengzhou University of Aeronautics, Zhengzhou, China; 2 College of Forestry, Henan Agricultural University, Zhengzhou, China; Guangdong Ocean University, CHINA

## Abstract

The objectives of this study were to comprehensively investigate the occurrence, distribution, and mobility of antibiotic resistant genes (ARGs) in the biofilm, water, and sediment from a section of the Weihe-river, in the northern Henan province, China. The abundances of nine ARGs belonging to four commonly used antibiotic classes (tetracyclines, sulfonamides, fluoroquinolones, and multidrug) and class 1 integron-integrase gene (intI1) were quantified. Sulfonamides gene (sulI) accounted for the highest percentage of detected ARGs in most sampling sites, including in water, biofilm, and sediment. Among the resistance genes, IntI1 and sul1 were significantly correlated (r>0.800, p<0.01) with a *fecal coliform* (FC) detected in the biofilm, and there was also a significantly positive correlation between the abundances of 16SrRNA and intI1 in the biofilms. Compared with the sediment and water samples, the biofilms contained sufficient nutrients to promote bacterial reproduction. Under sufficient total nitrogen and phosphorus concentrations, the horizontal gene transfer due to intI1 plays a key role in the formation and migration of ARGs within biofilms.

## Introduction

Antibiotics are widely used in controlling infectious diseases in humans and animals. They are also added to livestock feed as sub therapeutic and growth promoting agents. China is the world’s largest producer and consumer of antibiotics. Some studies have shown that in 2013, approximately 53800t of antibiotics were released into rivers and waterways in China [1]. The widespread use of antibiotics has led to their frequent detection in soil, surface water, groundwater, drinking water, and other environmental media. In addition to directly polluting the environment, antibiotics may induce the formation of antibiotic sensitive or resistant bacteria (ARB) and even antibiotic resistant genes (ARGs) in the environment [2]. ARGs not only remain in the environment but also migrate, transform, spread, and diffuse to other environmental media, eventually destabilizing the environment and entering the food chain, thereby affecting human health [3].

ARGs have gradually become a major threat to public health and environmental stability. To overcome this problem on a global and local scale, it is necessary to better understand the causes and mechanisms leading to the emergence and spread of ARGs. Recent studies have focused on the relationship between antibiotic residues and ARGs in water, sediment, and soil environments; however, the correlation between ARGs and biodiversity in the presence of a natural water biofilm is rarely studied [4,5,6,7]. A natural water biofilm is formed on the surfaces of rocks and sediments in rivers, lakes, and wetlands, composed of living components (algae, heterotrophic microorganisms) and non living components (inorganic minerals, organic polymers) [8]. The formation of biofilms on the underwater surface of river microbiota is significant for the functioning of river ecosystems. Although biofilms continuously regenerate biomass, they have a relatively fixed structure which can gather and concentrate suspended particles in water, including pollutants [9]. Natural biofilms affect the behavior of pollutants in water [10]. Reports on a direct analysis of ARGs in natural biofilm systems are lacking. Therefore, it is necessary to investigate the abundance and migration of ARGs in natural water biofilm, to make clear the transformation and mechanism of ARGs in antibiotic-contaminated ecosystems.

Weihe-river is a tributary of the Haihe River, which originates from Duhuo Town, Lingchuan County, Shanxi Province. The length of the river in Henan Province is 286 km, with a drainage area of 12911km^2^. Along the river basin in the northern Henan Province are important agricultural and industrial areas, and ecological problems are prominent [11].

In this research, the abundances of class 1 integron-integrase gene (intI1) and nine ARGs belonging to four commonly used antibiotic classes (tetracyclines, sulfonamides, fluoroquinolones, and multidrug) were quantified. The objectives were to comprehensively investigate the distribution and migration of ARGs in the biofilm, water, and sediment from the Weihe-river. We used the high throughput quantitative (PCR) and real-time quantitative PCR (qPCR) methods to detect the abundance and diversity of ARGs and explore the pollution level of ARGs. By analyzing the differences and correlations between the ARGs, integrons, and indicator microorganisms in the watershed, we provide clues on the spread of ARGs through water systems and a theoretical basis for their pollution control in the Weihe-river.

## Materials and methods

### Sample collection and sample properties

**Fig 1:**
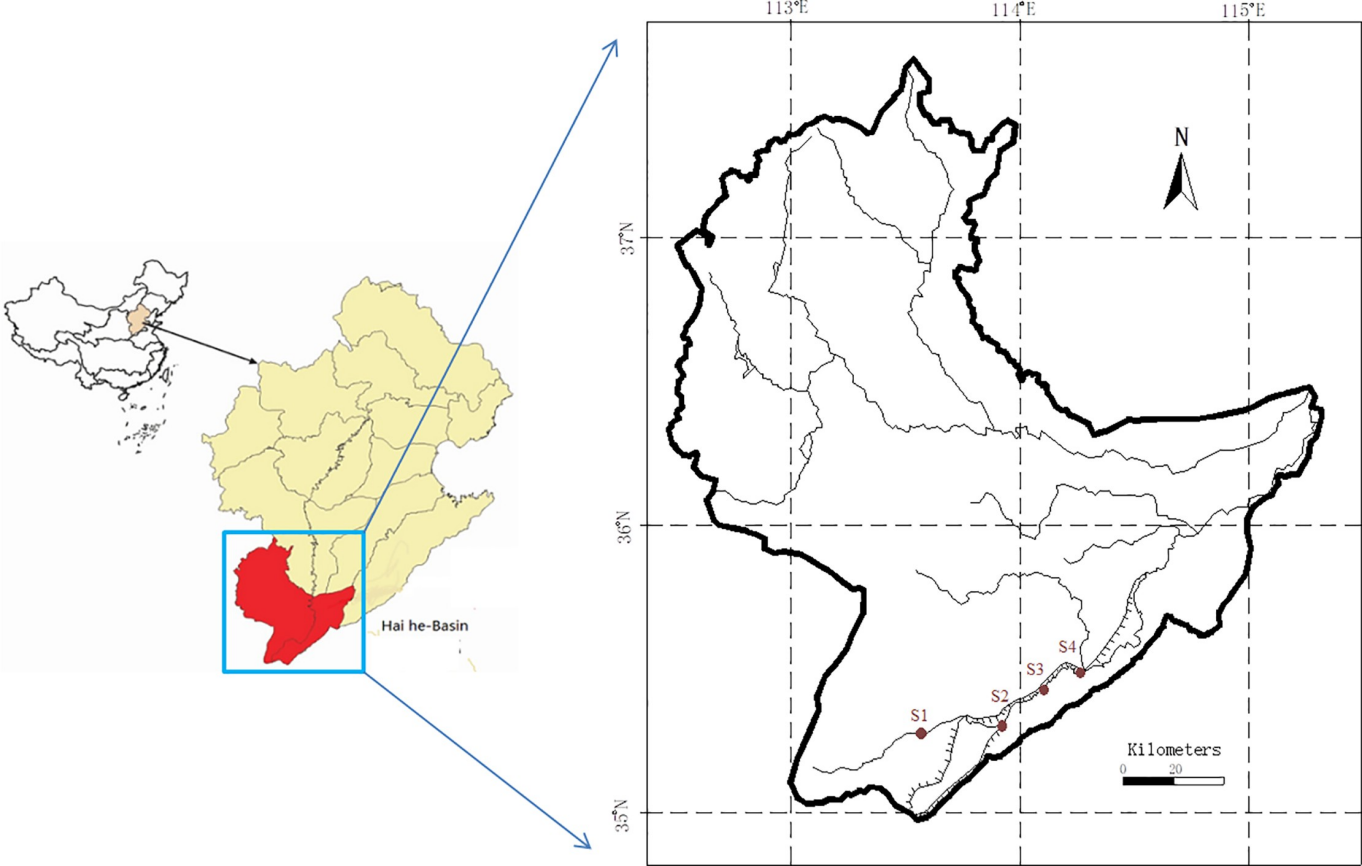
Sample stations (S1-S4) in the Weihe-river and its geographical location.

In July 2018, biofilm, sediment and water samples were collected in triplicates from the Weihe-river located in the north of Henan province, China, including from areas designated as S1 (upstream), S2 (urban area), S3 (town area), and S4 (village area) (Fig 1 and Table 1). The watershed was public areas and facilities. There are no regulatory restrictions on the collection of samples from this watershed for research purposes. No protected or threatened species or locations were involved in this study, and therefore no permission was required for samples collection in the field studies. The biofilm samples were obtained by scraping the surface of rocks or surface sediments at the sampling sites. The top 10 cm of the surface sediment samples with the biofilm removed were collected in stainless steel Containers, and the water samples from 5–10 cm over the collected sediment and biofilm were sampled. All samples were stored at −5°C in the dark, transported to a laboratory in 5 h, and processed instantly for antibiotic analysis and DNA extraction. The content of total organic carbon (TOC) in sediment and biofilm was determined through potassium dichromate–sulfuric acid digestion [12], and TOC in water was detected by a TOC analyzer (TOC-V CPH SSM 5000A, Shimadzu, Japan). Concentrations of the total nitrogen (TN) and total phosphorus (TP) in the biofilm and sediment were determined through high-temperature digestion with alkaline potassium persulfate [12].The TN concentration in the water samples was detected using the persulfate digestion method, and the TP concentration was detected using the potassium persulfate digestion method [12].The samples were analyzed in triplicates with relative standard deviations less than 10%.

**Table 1:** Sample stations along Weihe-river.

stations	Coordinates		Activities at sampling site
	longitude	latitude	
S1	113°40′02″	35°19′33″	Upstream
S2	113°52′46″	35°18′30″	Urban area with surface runoff
S3	114°03′55″	35°25′29″	County area with wastewater input
S4	114°16′59″	35°29′33″	Agriculture area with planting and breeding activities

### DNA isolation and analysis of ARGs

According to the manufacturer's instructions, the Axyprep bacterial genomic DNA micro preparation kit (American axygen) was applied to extract the total DNA in the biofilm and sediment samples, and the water DNA kit (omega bio-tek, USA) is used to extract the total DNA in the water. All the DNA samples were stored at low temperatures, and the DNA quality was examined with 1% agarose gel electrophoresis (Tanon 1600, China).

Concentrations of 9 target ARGs including 2 tetracycline resistance genes (tetA and tetO), 2 fluoroquinolone resistance genes (qnrA and qnrS), 3 sulfonamide resistance genes (sul1, sul2, and sul3), and 2 multidrug resistance genes (floR and cmlA) were detected in this study. In addition to the ARGs, a mobile genetic element such as the class 1integron gene (intI1) was also examined. qPCR was used to determine the abundances of target ARGs, intI1, and 16SrRNA gene in the samples. Detailed information on the primer sets designed in this study for PCR amplification can be found elsewhere [13] and they were listed in S1 Table.

DAN amplification reactions were carried out in a TB Green Premix Ex Taq^TM^ II (Tli RNaseH Plus) Kit (Takara, Bio Inc). For each real-time qPCR reaction, three replicates were performed in a 20.0 μL system, including 2×LightCycle 10.0μL SYBR® Premix Ex Taq^™^ II (Takara, Bio Inc), 0.4μL reverse primer and forward primer,1μL template DNA, 5.0 μL TB Green Premix Ex Taq^TM^ II (Tli RNaseH Plus) and 3μL sterile ddH_2_O. The positive control group and the negative control group did not produce any products in each reaction. The analysis process of qPCR reacting includes 30s at 95°C, 40cycles of 5s at 95°C, followed by 30s at 60°C and at 60°C extension for 10 min finally. The melting curve was generated by SmartChip PCR software, and threshold cycle (CT) value of 31 was set as the detection limit. The number of gene copies was defined as the average of the three replicates data obtained. The copy numbers of ARGs based on CT were calculated by application of comparative CT method [14]. The absolute abundances of the target ARGs and intI1 were calculated as follows [15, 16]:

$$C=10^{\left(31-C_{T})/(10/3\right)}$$ (1)

In the formula (1), *C* refers to the relative copy number of gene, and *CT* refers to the number of cycles during which PCR produces specific fluorescence.

$$A=\frac{C_{ARG}}{C_{16SrRNA}}\times A_{16SrRNA}$$ (2)

In the formula (2), *A* refers to the absolute abundance of an ARG, *C*_*ARG*_ refers to the relative copy number of the ARG, and *C*_*16SrRNA*_ refers to the relative copy number of 16SrRNA.

### Indicator bacteria analysis

The concentration level of indicator bacteria reflects the microbial pollution in the watershed. *Escherichia coli* (EC) and *Enterococcus* (ENT) are microorganisms adopted by the World Health Organization and European Union as indicators, while *fecal coliform* (FC) is commonly used in China. FC was studied as indicator organisms in this study. The samples were transported to the laboratory by cold storage and processed within 5h.The quantification of FC in water was conducted according to Environmental protection standard of the people's Republic of China(HJ 347.2–2018) [17], and the concentration of FC is reported in colony forming units per 1L (CFU / L). The membrane filtration technique was used for quantification of FC in sediment and biofilm samples [18]. 1.0 g wet weight of sediment (biofilm) sample was added 100 ml of sterile saline water, and then shake at 100 rpm for 45s. Two 10-ml supernatant were collected for analysis. Appropriate dilutions were chosen according to the technique used for water samples, and bacteria data in this study are reported on a CFU/L basis.

### Statistical analysis

The data were organized in Microsoft Excel 2010. IBM SPSS Statistics 25 was used for data analysis and statistical difference calculation. Heatmaps illustrations were generated by Heatmapper, which is a freely available web server. The data were expressed in terms of the average ±SD (standard deviation). The correlations with a significance level below 0.05 were defined as statistically significant.

## Results and discussion

### ARGs in Weihe-river ecosystem

**Fig 2:**
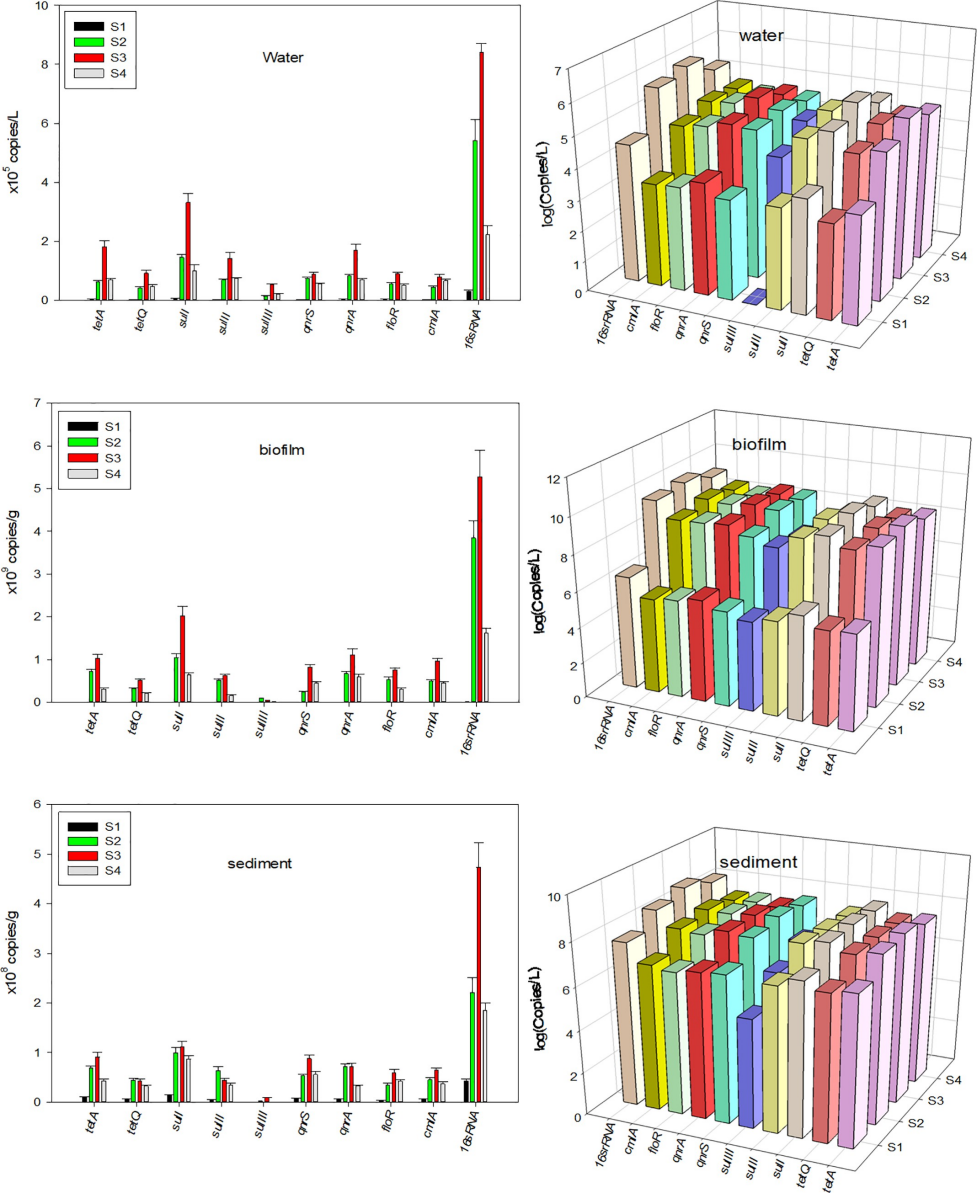
Compositional patterns of antibiotics in individual water samples, biofilm samples and sediment samples collected from the Weihe-river.

Nine target genes including tetracycline-related ARGs (tetA and tetQ), fluoroquinolone-related ARGs (qnrS and qnrA), fluoroquinolone-related ARGs (sulI, sulII, and sulIII), and multidrug-related ARGs (floR and cmlA) were detected in the Weihe-river. The detection frequency of 8 genes was 100%, including tetA, tetQ, qnrS, qnrA, sulI, sulII, floR, and cmlA. The target antibiotics were frequently detected in all the samples, indicating the widespread distribution of antibiotics in this watershed. Among the four tested sites along the river, the total abundances of ARGs at S2, S3, and S4 were significantly higher than that at S1. Different land use patterns in the basin may have led to different ARG concentrations. Fig 2 shows an overview of the absolute abundance of ARGs in the water, biofilm, and sediments from the Weihe-river. The absolute abundance of ARGs in the sample shows the following decreasing pattern: biofilm > sediment > water. Meanwhile, the average abundances of ARGs from different sampling sites decreased as follows: S3 >S4 > S2 > S1 for the water samples, S3 >S2 > S4 > S1 for the biofilms, and S3 > S4 ≈ S2 > S1 for the sediments. The results showed that ARGs abundance changed with the degree of urbanization, and land use maybe the main factor affecting the existence of resistance genes in Weihe-river watershed. At S2 and S3, the increase of ARGs concentration in biofilm and sediment samples may be caused by wastewater or domestic sewage runoff from wastewater treatment plants (WWTP). In the case of S4, this could be attributed to planting and breeding activities. Studies have shown that the more frequent human activities in the region, the higher content of resistance genes in environmental media. Specifically, impervious surface ratios, wastewater from wastewater treatment plants, and land use practices for aquaculture have been considered main sources of ARGs in freshwater systems [19].

Fig 2 shows the absolute abundances of ARGs in water samples, biofilm samples and sediment samples collected from the Weihe-river. Most of the detected ARGs were present in all the samples, showing that the resistant genes pollution in the watershed was significant. sulI accounted for the highest percentage of detected ARGs in most sampling sites, with concentrations ranging from 5.40×10^3^ to 3.31×10^5^ copies/L, 7.50×10^5^ to 2.01×10^9^ copies/g, 1.46×10^5^ to 1.12×10^8^ copies/g in the water, biofilm, and sediment samples, respectively. Two multidrug resistance genes (floR and cmlA) were highly diverse and abundant in the watershed, with an average abundance of 9.74×10^4^copies/L in water, 8.76×10^8^ copies/g in the biofilm, and 7.34×10^7^ copies/g in the sediment. Previous research has shown that tet(A) is the most prevalent tetracycline resistance in most rivers [20,21]. In the current study, the tet (A) gene was sporadically detected in the water and biofilm samples. The absolute abundances of the nine ARGs at the different sampling sites showed the following decreasing order: sulI> tetA> sulII> qnr(A) > qnr(S) > floR> cmlA> sulIII> tetQ in water, sul I> qnr(A) > tetA> cmlA> floR> qnr(S) > sulII> tetQ> sulIII in the biofilms, and sulI> tetA> qnr(S) > qnr(A) > cmlA> sulII> floR> tetQ> sulIII in the sediments. The quantitative analysis of 16SrRNA gene can help to assess the abundance of bacteria in the sample, and can also be applied to standardize the abundance of ARGs in watershed. Absolute abundance of 16S-rRNA gene copies fluctuated from 3.04×10^4^ to 8.41×10^5^ copies/L in water, 2.11×10^7^ to 5.27×10^9^ copies/g in the biofilm and 4.31×10^7^ to 4.73×10^8^ copies/g in the sediments. Zhao (2020) reported similar levels of 16S-rRNA gene (9.06×10^6^ to 2.93×10^8^ copies/L) in sediment from Haihe Estuary [22]. Compared with other river sediments, 16S-rRNA gene concentrations were lower than those found in river from Zhuhai city [4], and urban river from Beijing city [23], while higher than that in the Jiulong River in southeast China [24].

**Fig 3:**
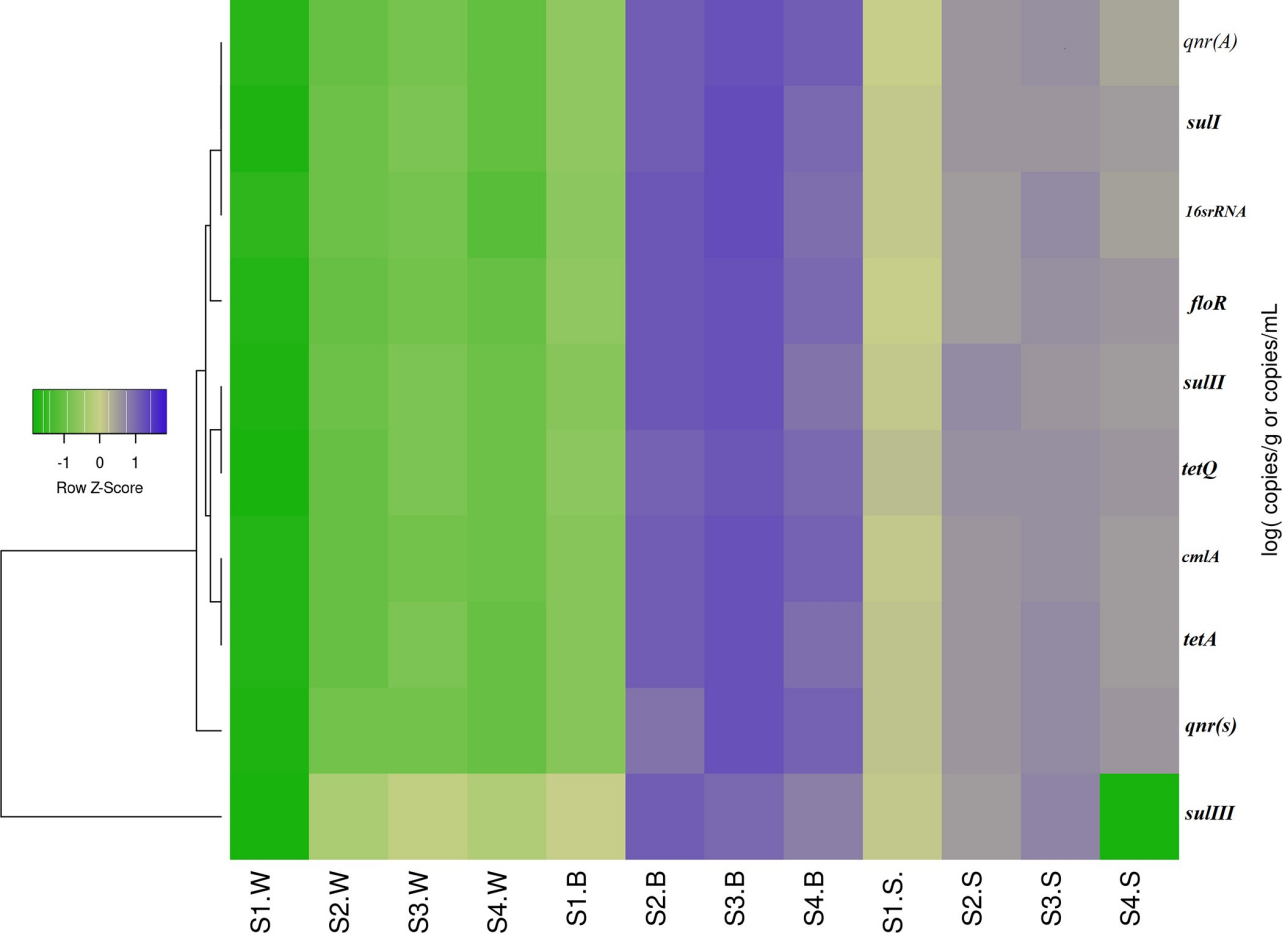
Heatmap of the abundances of the ARGs in biofilm (S1-4.B), sediment (S1-4.S and water (S1-4.W) of the Weihe-river. Blue indicates higher abundance of ARGs detected, while green indicates lower abundance of ARGs detected.

Through the heatmap analysis (see Fig 3), we can understand the correlation between the resistance genes and 16SrRNA and reveal their distribution patterns in the different media. Significant correlations between the absolute abundances of sulI /16SrRNA and qnrA /16SrRNA were observed in all the samples (p< 0.05). Moreover, the absolute abundance of tetA exhibited a strong correlation with cmlA (p< 0.01), indicating that these ARGs had a mutual relationship and could coordinate the response of antibiotics to corresponding bactericidal effects [25].

### Migration of ARGs in the watershed

The mobility of ARGs is closely related to integrons. Integronsare a type of mobile DNA molecule systems with a special structure, which can capture and integrate exogenous genes, particularly ARGs, so that they can be transformed into expression units of functional genes and then horizontally transmitted in bacteria through transposons and plasmids [26,27]. In the current study, the most common integron 1 (intI1) was selected for monitoring and analysis. In general, intI1 was ubiquitous in Weihe-river, and a slightly lower abundance was observed in the sediments (2.51×10^5^to 3.74×10^7^copies/g) than in the biofilms (4.41×10^5^ to 6.48×10^8^ copies/g). Their abundance in water ranged from 2.27×10^4^ to 4.66×10^7^copies/ L. The results show that the pollution level of intI1 in water environment is serious. To explore the effect of horizontal gene transfer on the absolute abundance of antibiotics in water body, a Bayes correlation analysis was used to evaluate the relationships betweenintI1, 16SrRNA, and selected chemical parameters. As evident from Table 2, a significant positive correlation is observed between the abundances of intI1 and 16SrRNA in the biofilms (r = 0.822, p < 0.01). Similar results were also observed in the case of water (r = 0.784, p< 0.01). Compared with the sediments and water, the biofilms contained sufficient nutrients to promote bacterial reproduction. Under sufficient TN and TP, the horizontal gene transfer due to intI1 plays an important role in the formation of ARGs within biofilms.

**Table 2:** Correlation analyses of intI1, 16SrRNA, and environmental factors in Weihe-river.

**water**	*intI1*	*16SrRNA*	*TN*	*TP*	*TOC*
intI1	1				
16SrRNA	0.784*	1			
TN	0.320	0.564	1		
TP	-0.113	0.028	0.561	1	
TOC	0.337	0.512	0.803**	0.433	1
**biofilm**	*intI1*	*16SrRNA*	*TN*	*TP*	*TOC*
intI1	1				
16SrRNA	0.822**	1			
TN	0.585	0.576	1		
TP	0.774*	0.411	0.368	1	
TOC	0.422	0.468	0.958**	0.219	1
**sediment**	*intI1*	*16SrRNA*	*TN*	*TP*	*TOC*
intI1	1				
16SrRNA	0.611	1			
TN	-0.373	-0.324	1		
TP	0.801**	0.459	-0.388	1	
TOC	0.064	0.318	-0.520	0.315	1

* Significant at p < 0.05

** significant at p < 0.01.

### Correlation between ARG and indicator bacteria

**Fig 4:**
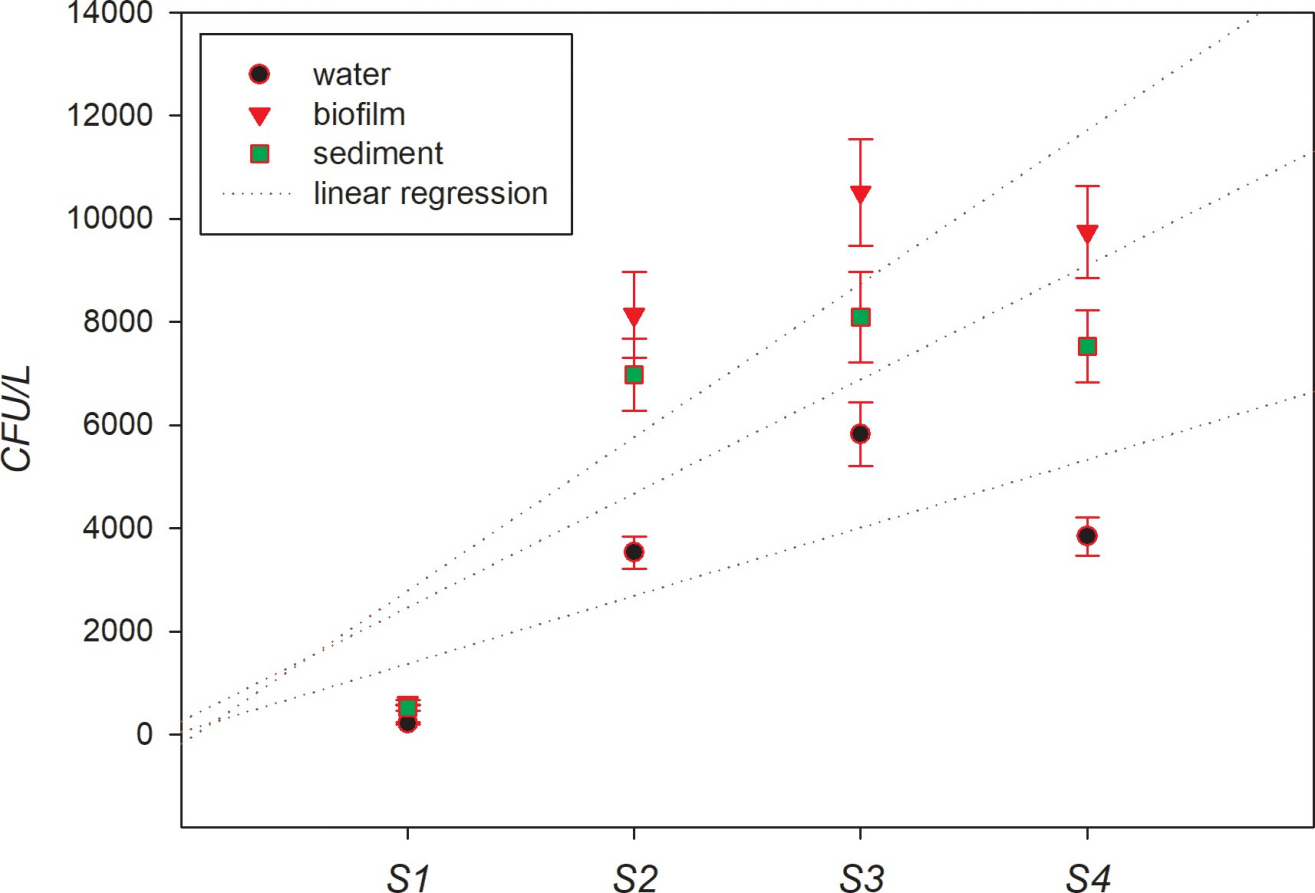
Concentration of *fecal coliforms* (FC) in samples from Weihe-river.

Intestinal bacteria excreted into the environment may promote the horizontal transfer of ARGs with indigenous bacteria, leading to the spread of ARGs in the environment [28]. In this study, intestinal bacteria were used as indicator bacteria to explore their relationship with the resistance genes. Fig 4 shows the ephemeral nature of *fecal coliform* (FCs) pollution at each sampling point in the Weihe-river. There are significant differences in the occurrence of FCs at different points. Site 3 is the most consistently polluted area, with average concentrations as highas9.82×10^2^ CFU/L, 8.09×10^3^CFU/L, and 9.15×10^3^CFU/Lin water, biofilm, and sediment, respectively. There is no significant correlation between the FC in water/sediment and water/biofilm, indicating that the presence of FCs in biofilms and sediments is not always an accurate indicator of FCs count in water, it also depends on rainfall and other human activities in the basin. To identify the quantitative correlation between ARGs and the FCs, a Pearson correlation analysis was carried out between FCs and the absolute abundances of ARGs (see Table 3). The results showed that among the nine resistance genes, sul1 were significantly correlated (r>0.800, P<0.01) with the FCs in the biofilm, while the other genes were weakly correlated with the indicator bacteria. This shows that indicator bacteria may play a key role in the spatial occurrence and spread of antibiotic resistant genes. Therefore, it is necessary to study the relationship between intestinal flora and resistance genes, particularly betweensul1and FC. This can be attributed to the fact that IntI1 and sul1 can transfer genes horizontally among different microbial individuals through movable genetic elements (such as plasmids, transposons, integrators, and insertion sequences) [29].

**Table 3:** Pearson correlation between indicator microorganisms and ARGs.

	*tetA*	*tetQ*	*sul1*	*sulII*	*sulIII*	*qnrS*	*qnrA*	*floR*	*cmlA*	*16SrRNA*	*IntI1*
*water*	0.242	-0.356	0.813**	0.612	0.464	0.114	0.087	-0.221	-0.153	0.721*	0.886**
*biofilm*	0.221	0.012	0.876**	0.744*	0.011	-0.146	0.267	0.526	0.722*	0.773*	0.781*
*sediment*	0.351	0.217	0.741*	0.726*	0.543	0.085	0.101	0.443	0.637	0.682*	0.681*

* Significant at p < 0.05

** significant at p < 0.01.

## Conclusions

We investigated the occurrence, distribution, and mobility of 9 ARGs in the biofilm, water, and sediment samples collected from the Weihe-river. The detection frequency of eight genes was found to be 100%, including tetA, tetQ, qnrS, qnrA, sulI, sulII, floR, and cmlA. The abundances of the 9antibiotic resistant genes in watershed followed decreasing order as: biofilm > sediment > water. The target antibiotics were frequently detected in all the samples, indicating their widespread distribution in this watershed. Among the four sites along the river, the total abundances of ARGs at S2, S3, and S4 were significantly higher than that at S1. Different patterns of land utilizations in the basin may have led to different ARG concentrations. Significant correlations between the absolute abundances of 16SrRNA/sulI, 16SrRNA/qnr(A), and 16SrRNA/intI1 were observed in the biofilm samples (p<0.01). Furthermore, among the nine resistance genes, sul1 were significantly correlated (r>0.800, p<0.01) with the FC in the biofilm and the selected chemical parameters. Owing to the complexity of ARG/ intI1/fecal coliform co-occurrence in river biofilm communities, we concluded that biofilms has a strong ability to accumulate and store resistance genes compared to water and sediments at the sampling sites along the Weihe-river.

## Supporting information

S1 Table Primer systems used in this study. (DOCX)

S2 Table Summary data of the physical and biological parameters measured in this study. (DOCX)
