# Supplementary material for: Prevalence and pollution characteristics of antibiotic resistant genes in one high anthropogenically-impacted river
Source: PLoS One. 2020 Apr 9;15(4):e0231128. doi: 10.1371/journal.pone.0231128 (PMC7145097; doi:10.1371/journal.pone.0231128)
Supplement: S1 Table — (DOCX) [file pone.0231128.s001.docx]

Table S1. Primer systems used in this study

| Target gene | primers | sequence[5'-3'] | amplicon/bp | Annealing temp (℃) |
| --- | --- | --- | --- | --- |
| tetA | tetA-F | GCTACATCCTGCTTGCCTTC | 210 | 57 |
|  | tetA-R | CATAGATCGCCGTGAAGAGG |  |  |
| tetQ | tetQ-F | AGAATCTGCTGTTTGCCAGTG | 169 | 63 |
|  | tetQ-R | CGGAGTGTCAATGATATTGCA |  |  |
| sulI | sul1-F | CACCGGAAACATCGCTGCA | 158 | 60 |
|  | sul1-R | AAGTTCCGCCGCAAGGCT |  |  |
| sulII | sulII-F | CTCCGATGGAGGCCGGTAT | 190 | 56 |
|  | sulII-R | GGGAATGCCATCTGCCTTGA |  |  |
| sulIII | sulIII-F | TCCGTTCAGCGAATTGGTGCAG | 128 | 55 |
|  | sulIII-R | TTCGTTCACGCCTTACACCAGC |  |  |
| qnrS | qnrS-F | GTGAGTAATCGTATGTACTTTTGC | 169 | 58 |
|  | qnrS-R | AAACACCTCGACTTAAGTCT |  |  |
| qnrA | qnrA-F | TTCTCACGCCAGGATTTG | 521 | 60 |
|  | qnrA-R | CCATCCAGATCGGCAAA |  |  |
| floR | floR-F | GTCATTCCTCACCTTCATCCTAC | 243 | 60 |
|  | floR-R | GACACCAGCACTGCCATTG |  |  |
| cmlA | cmlA-F | GTTGGCGGTACTCCCTTGCC | 240 | 55 |
|  | cmlA-R | GGCCACCTCCCAGTAGAACG |  |  |
| int1 | int1-F | CGCTTCGTGATGCCTGCTT | 146 | 57 |
|  | int1-R | CATTCCTGGCCGTGGTTCT |  |  |
| 16SrRNA | 16SrRNA-F | TCCTACGGGAGGCAGCAGT | 466 | 60 |
|  | 16SrRNA-R | GGACTACCAGGGTATCTAATCCTGTT |  |  |
