# Supplementary material for: Prevalence and pollution characteristics of antibiotic resistant genes in one high anthropogenically-impacted river
Source: PLoS One. 2020 Apr 9;15(4):e0231128. doi: 10.1371/journal.pone.0231128 (PMC7145097; doi:10.1371/journal.pone.0231128)
Supplement: S2 Table — (DOCX) [file pone.0231128.s002.docx]

Table S2. Summary data of the physical and biological parameters measured in this study.

|  |  | intI1 | TP | TN | TOC | FC |
| --- | --- | --- | --- | --- | --- | --- |
|  |  |  |  |  |  |  |
| water | S1 | (2.27±0.14)╳10^4^ copies/L | 0.22±0 mg/L | 1.58±0.11 mg/L | 2.37±0.15 mg/L | 1.56╳10^2^ CFU/L |
|  | S2 | (3.46±0.24)╳10^7^ copies/L | 1.94±0.21 mg/L | 8.77±0.63 mg/L | 22.37±2.27 mg/L | 7.52╳10^2^ CFU/L |
|  | S3 | (4.71±0.35)╳10^7^ copies/L | 3.87±0.40 mg/L | 12.45±1.33 mg/L | 31.78±2.86 mg/L | 9.82╳10^2^ CFU/L |
|  | S4 | (3.97±0.42)╳10^7^ copies/L | 3.02±0.23 mg/L | 10.55±1.11 mg/L | 30.42±2.78 mg/L | 8.76╳10^2^ CFU/L |
| biofilm | S1 | (4.41±0.37)╳10^5^ copies/g | 16.7±1.22 mg/kg | 103.31±12.12 mg/kg | 2.55±0.19 % | 1.12╳10^3^ CFU/L |
|  | S2 | (6.48±0.54)╳10^8^ copies/g | 311.44±29.45 mg/kg | 841.22±88.53 mg/kg | 4.67±0.34 % | 6.21╳10^3^ CFU/L |
|  | S3 | (5.79±0.61)╳10^8^ copies/g | 287.22±30.14 mg/kg | 1032.2±100.78 mg/kg | 5.41±0.47 % | 8.09╳10^3^ CFU/L |
|  | S4 | (5.21±0.47)╳10^8^ copies/g | 332.35±28.65 mg/kg | 942.17±87.65 mg/kg | 5.02±0.42 % | 6.45╳10^3^ CFU/L |
| sediment | S1 | (2.51±0.19)╳10^5^ copies/g | 57.4±3.77 mg/kg | 19.21±2.10 mg/kg | 1.21±0.14 % | 1.07╳10^3^ CFU/L |
|  | S2 | (2.17±0.22)╳10^7^ copies/g | 432.63±50.62 mg/kg | 137.82±11.79 mg/kg | 3.42±0.28 % | 8.21╳10^3^ CFU/L |
|  | S3 | (3.74±0.27)╳10^7^ copies/g | 502.24±46.45 mg/kg | 747.82±68.44 mg/kg | 3.54±0.31 % | 9.15╳10^3^ CFU/L |
|  | S4 | (2.97±0.22)╳10^7^ copies/g | 421.33±39.76 mg/kg | 698.62±63.56 mg/kg | 2.63±0.22 % | 6.27╳10^3^ CFU/L |
